# Impact of Air Pollution (PM_2.5_) on Child Mortality: Evidence from Sixteen Asian Countries

**DOI:** 10.3390/ijerph18126375

**Published:** 2021-06-12

**Authors:** Asim Anwar, Inayat Ullah, Mustafa Younis, Antoine Flahault

**Affiliations:** 1 Department of Management Sciences, COMSATS University Islamabad, Attock Campus Kamra Road, Attock 43600, Pakistan; 2 Department of Government and Public Policy, National University of Science & Technology, Islamabad 44000, Pakistan; inayat@s3h.nust.edu.pk; 3 Department of Health Policy & Management, Jackson State University, Jackson, MS 39217, USA; Younis99@gmail.com; 4 Swiss School of Public Health (SSPH+), 8001 Zurich, Switzerland; Antoine.Flahault@unige.ch; 5 Institute of Global Health, Faculty of Medicine, University of Geneva, 8001 Zurich, Switzerland

**Keywords:** air pollution, child mortality, economic growth, Asian countries

## Abstract

Air pollution in Asian countries represents one of the biggest health threats given the varied levels of economic and population growth in the recent past. The quantification of air pollution (PM_2.5_) vis à vis health problems has important policy implications in tackling its health effects. This paper investigates the relationship between air pollution (PM_2.5_) and child mortality in sixteen Asian countries using panel data from 2000 to 2017. We adopt a two-stage least squares approach that exploits variations in PM_2.5_ attributable to economic growth in estimating the effect on child mortality. We find that a one-unit annual increase in PM_2.5_ leads to a nearly 14.5% increase in the number of children dying before the age of five, suggesting the severity of the effects of particulate matter (PM_2.5_) on health outcomes in sixteen Asian countries considered in this study. The results of this study suggest the need for strict policy interventions by governments in Asian countries to reduce PM_2.5_ concentration alongside environment-friendly policies for economic growth.

## 1. Introduction

Air pollution has been considered a major environmental threat to global health in recent years. The World Health Organization (WHO) in 2018 claimed that nine out of ten people breathe polluted air, and more importantly, air pollution is responsible for 7 million deaths each year globally. Among all the air pollutants, fine particulate matter (diameter less than 2.5 microns, PM_2.5_) is one of the most severe threats to human health [1,2]. PM_2.5_ includes precarious airborne chemicals and particles with multiple forms and sources. The most hazardous of these sources are human activities, such as nitrogen dioxide from vehicles, sulfur dioxide from power plants, and ground-level ozone, as well as particulate matter. The last type is important due to its ability to penetrate into peoples’ lungs and enter their bloodstream [3,4]. Epidemiological researchers have substantiated that these health impacts are induced by continuing ambient PM_2.5_ concentrations and the coherent risk factors that vary from country to country [5,6].

Studies have rated PM_2.5_ as the most lethal of the particulate matters, in association with child mortality [7,8], given that growth-stage of infants (0–1 years) and children under five are more susceptible to poor air quality [3]. Children are relatively more exposed to air pollution than adults due to longer engagement in outdoor activities and mouth breathing, which increases their intake of air pollutants [9]. The WHO (2018) reported 543,000 deaths in children under 5 years of age and 52,000 deaths in those aged 5–15 due to ambient air pollution in 2016, which mostly occurred in low- and middle-income countries. Although the mortality rate of children under five has declined from 19.6 million in 1950 to 5.4 million in 2017, developing countries are still far away from developed countries in overcoming child mortality [10]. Asian countries are more prone to this threat, as 92% of the Asia and Pacific population are exposed to levels of air pollution that constitute a significant risk to their health (UN, 2019).

Asian countries are experiencing increasing levels of air pollution due to fast industrialization, urbanization, coal power plants, transportation, population growth and household solid fuel use [8,11]. The Asian region has become notorious for reported vulnerability to higher mortality (59% of total global deaths) due to low quality air [12,13,14]. The state of global air report claimed that the countries with the highest mortality rate due to air pollution were in Asia, and includes India, China, Pakistan, Bangladesh and Indonesia. The Asian countries with the highest mortality rate includes Bangladesh, India, Pakistan, Myanmar, Nepal, Philippines, Mongolia, China, Iran and Vietnam, with 38.3, 50.8, 84.7, 49.8, 46.0, 16.5, 13.2, 7.7, 10.2 and 12.6 (deaths per 100,000 (<5 years)), respectively, in the year 2016 (WHO, 2018), while the countries with moderate rates of mortality are Thailand (4.0), Turkey (2.7), Malaysia (1.4), Russia (1.3) and Sri Lanka (1.3). The PM_2.5_ exposure levels (micrograms per cubic meter) in the studied countries are Bangladesh (53.2), India (65.2), Pakistan (55.2), Myanmar (34.7), Nepal (94.3), Philippines (18.4), Mongolia (40.4), China (49.2), Iran (35.1), Vietnam (29.7), Thailand (26.2), turkey (42.0), Malaysia (16.0), Russia (13.7) and Sri Lanka (15.2), for the year 2016 (WHO, 2018).

Several studies have shown a positive relationship between air pollution (PM_2.5_) and child mortality in recent years. For example, Lien et al. and Owili et al. [15,16] showed a positive relationship of air pollution (PM_2.5_) with child mortality in those under five and maternal deaths in 45 Asian countries and Africa. Gouveia et al. [9] evaluated the impact of air pollution on child mortality in four Latin American cities (Mexico city, Santiago, Chile and Rio de Janeiro) using the generalized additive model with Poisson regression. The result suggests the positive relationship between air pollution and child mortality for these cities. Sarkodie et al. [17] examined the association between ambient air pollution (PM_2.5_), mortality and life expectancy in 54 countries, including Europe and North America, using time series data from 2000–2016. The study applied a generalized least square (GLS) random effect model with first-order autoregressive disturbance, which revealed a significant and positive association among ambient air pollution, mortality and life expectancy in the studied countries. The long-term health related assessment of PM_2.5_, mostly undertaken in developed countries, showed the positive association between PM_2.5_ and mortality over the years [18,19]. However, despite experiencing the worst air pollution and its impact on child mortality, there are limited cross-country studies available on Asian countries that examine the relationship between air pollution (PM_2.5_) and child mortality in those under five [15].

This study contributes to the literature in multiple aspects. Firstly, it takes into account the potential income effect of PM_2.5_ when explaining its impact on child mortality. We do this because of the potential endogeneity in PM_2.5_ when estimating its health effect through a reduced-form equation. Secondly, we use cross-country panel data from 2000 to 2017 for sixteen Asian countries, which allow us to apply country fixed effect (CFE) and year fixed effect (YFE) to address any omitted time-invariant and country-specific characteristics when estimating the effect of PM_2.5_ on child mortality. Thirdly, we selected Asian countries for two reasons. First, health issues, particularly in children under five, have been observed at high rates in Asian countries, including the populous countries India, Pakistan, Bangladesh and Indonesia [14]. Second, over the last twenty years, some Asian countries, such as China, Malaysia, Turkey and Russia, have adopted large-scale pollution mitigation measures, such as investment in wind and solar power, caps on coal use, and the use of filters and scrubbers [20,21]. Therefore, the purpose of this study is to examine the effect of PM_2.5_ on child mortality in Asian countries, thus confronting the serious environmental issues that have arisen in recent years. The following section explains the data, methods and empirical specification. Results are presented in the Section 3, followed by discussion and implications in the Section 4. The Section 5 concludes.

## 2. Materials and Methods

We follow the health production function proposed by [22], which is shown below:

(1) H=h(d, b)

(2) D=d(e,a)

(3) H=h(e,a,b)

In Equation (1), *H* represents health status, which is a function of the level of pollution exposure or dose (*d*) and mitigating activities (*b*). The pollution exposure, as represented by *D* in Equation (2), further depends on averting activities denoted by (*a*) and the concentration of pollutants (*e*). Averting behavior is the attitude of shunning a certain stimulus, whereas mitigating behavior involves studying the advent and propagation of a certain disorder and finding measures to root out its occurrence. In the reduced form, Equation (1) can be written as Equation (3), where d is substituted with (*e,a*). This reduced-form equation allows us to determine the indirect effect of averting behavior and pollutant concentration on the health status of individuals. In aggregate terms, however, the health production function can be measured through mortality rate (Thornton, 2002). Although the dose–response function yields weaker estimates for pollution reduction, this model is the only option that can be used to deal with aggregate data from large entities, such as countries and states, for which the implicit cost of pollution cannot be easily measured. Considering the aggregate health production function, the reduced-form Equation (3) becomes a dose–response function, with pollution and mortality as its key indicators and excluding the averting and mitigating costs (difficult to accurately measure) as given by Equation (4). Estimates of reduced forms such as Equation (4) or Equation (5) are useful both in providing policy-relevant parameters and in predicting the relationship between health and health-related inputs.

(4) H=h(e) 

Controlling for physical and socio-economic characteristics is important in the estimation of health production function, as suggested by [23]. The reduced-form equation for child mortality is consistent with findings obtained in earlier studies that point to the fact that families with relatively high incomes and education tend to spend more on health. In this study, we follow the proposed framework in analyzing the health production function via the aggregate data on the countries, as below:

(5) E=e(y, z)

where *E* is determined by economic development (*y*) and other components of pollution, such as population density and education. By incorporating Equation (5) in Equation (4), the health production function (4) is derived in Equation (6):

(6) H = h(e(y, z)) 

We first empirically estimate Equation (6) through the following reduced-form specification:

(7) $$\ln{\left(Child\;Mortality\right)}_{it}=\alpha_{0}+\alpha_{1}PM2.5_{it}+\alpha_{2}{\left(GDP\right)}_{it}+\alpha_{3}{\left(GDP\right)}_{it}^{2}+\alpha_{4}{\left(POP\right)}_{it}+\alpha_{5}{\left(POP\right)}_{it}^{2}+\alpha_{6}Edu_{it}+\gamma_{i}+\varepsilon_{it}$$

where the log of child mortality represents child mortality in those under five in country *i* in year *t*. PM_2.5_ represents mean annual exposure (micrograms per cubic meter) in country *i* in year *t*. Income is represented by per capita gross domestic product $\left(GDP_{it}\right)$ and its square ${\left(GDP\right)}_{it}^{2}$ in country *i* in year *t*. Population density $\left(POP_{it}\right)$ is measured in terms of persons per square km in country *i* in year *t*. We used the square of GDP and the square of population density because, as found by a number of other studies (e.g., environmental Kuznets curves), PM_2.5_ has a non-linear relationship with income and population density. In other words, as income increases, the studies show, PM_2.5_ increases to begin with. However, as income increases to a certain threshold, pollution starts decreasing, indicating the mitigating behavior of individuals towards pollution after they achieve a certain income level. The same is true for population density. $\gamma_{i}$ represents the individual country-specific time invariant characteristics controlled through the fixed effect model. The $\varepsilon_{it}$ is the error term clustered at the country level. The Asian countries included in this study on the basis of data availability are Bangladesh, China, India, Indonesia, Iran, Malaysia, Mongolia, Myanmar, Nepal, Pakistan, Philippines, Russia, Sri Lanka, Thailand, Turkey and Vietnam.

In Equation (7), the PM_2.5_ is considered endogenous because of the potential reverse causality of PM_2.5_ with child mortality. Since our sample of Asian countries includes middle-income economies such as Russia, Malaysia, Turkey and China, we suspect that increased child mortality will incite government responses through pollution control measures in big cities. Earlier studies, such as [24], also point out the potential endogeneity of PM_2.5_ in Asian countries. Our reduced-form results shown in Table 1 also confirm t instrument’s validity e.g., after controlling for population and the square of population and other covariates, the coefficients are jointly insignificant. We therefore adopt alternate specifications in our two-stage least square (2SLS) model by taking income and the square of income as instruments to estimate the first stage of variation in PM_2.5_. A number of studies have suggested the existence of a non-linear relationship between income and environment [25,26]. We exploit this variation in PM_2.5_ attributable to variation in income level measured in terms of GDP per capita in Asian countries. Our first-stage specification estimates the endogenous PM_2.5_ through the following:

(8) $$\hat{PM2.5_{it}}=\alpha_{0}+\alpha_{1}{\left(GDP\right)}_{it}+\alpha_{2}{\left(GDP\right)}_{it}^{2}+\alpha_{3}{\left(POP\right)}_{it}+\alpha_{4}{\left(POP\right)}_{it}^{2}+\gamma_{i}+\varepsilon_{it}$$

where $\hat{PM2.5_{it}}$ in country *i* in year *t* is estimated by income measured in terms of per capita gross domestic product $\left(GDP_{it}\right)$ and its square ${\left(GDP\right)}_{it}^{2}$ in country *i* in year *t*, and the population density $\left(POP_{it}\right)$ measured in terms of persons per square km in country *i* in year *t*. The $\varepsilon_{it}$ is the error term clustered at the country level. The effect on the child mortality is estimated by the second-stage equation below.

(9) $$\ln{\left(Child\;Mortality\right)}_{it}=\beta_{0}+\beta_{1}{\hat{PM2.5}}_{it}+\beta_{2}Edu_{it}+\gamma_{i}+V_{it}\;$$

where child mortality in country *i* in year *t* is estimated by the estimated value of PM_2.5_ in the first stage. Our first-stage Equation (8) hypothesizes a U-shaped relationship between income and environment, which is consistent with earlier studies, such as [26].

The study uses child mortality under five as a dependent variable, the data for which is retrieved from the World Health Organization (WHO) Global Health Observatory repository and World Bank tables as shown in Table A1 in the Appendix A. The two datasets are included in the study to compare the results collected individually by the two different organizations. The World Bank defines child mortality as the probability of a child born in a specific year or period dying before reaching the age of 5 years, if subject to age-specific mortality rates of that period, expressed per 1000 live births. The data from WHO repository measures the prematurity deaths as the annual number of deaths due to specific cause among children less than five years. The data on air pollution (PM_2.5_) measured as mean annual exposure (micrograms per cubic meter), income (GDP per capita), average education (school enrollment, secondary (% gross)) and population density (persons per km^2^) are taken from the World Bank dataset for the period from 2000 to 2017 for sixteen Asian countries.

## 3. Results

Table 1 shows the reduced-form relationship between PM_2.5_ and child mortality in sixteen Asian countries. We find no significant effect of income and the square of income on child mortality under five after controlling for population density, the square of population density and education covariates, which suggests the endogeneity of PM_2.5_ as an explanatory variable. Throughout our regressions, we used country fixed effect to overcome any time-invariant, country-specific differences in the outcome variables.

In the first stage, by taking income (GDP per capita) and the square of it as instruments, we find significant variation in the endogenous variable (PM_2.5_) after controlling for the population covariates reported in Table 2. Thus, the first-stage results validate the use of PM_2.5_ as an endogenous variable to predict the indirect effect of income on the child mortality in sampled countries, as reflected by the F-test value of 15.85.

The results in Table 2 show negative effect of GDP per capita on PM_2.5_ and the positive effect of the square of GDP per capita on PM_2.5_, which suggests the existence of a U-shaped non-linear relationship in the aggregate data. Column (1) in Table 2 shows the simple OLS effect, while column (2) and column (3) show the fixed effect and random effect results, respectively. The application of the country fixed effect increases the significance level from 10% to 1%; however, it decreases the economic significance, as observed by the small values of the coefficient. Since our concern in the first stage is only to utilize the significance level (not the coefficients), we use the results of the fixed effect in the second-stage estimation.

This is surprising, since the sampled Asian countries have low pollution at the early stage of development, and high pollution at the later stages. One explanation for this U-shaped non-linearity could be the variation in the current economic development across these countries. For example, Russia, China, Turkey and Malaysia are categorized as upper-middle-income countries, while Pakistan, India and Bangladesh, etc., are lower-middle-income countries. We utilize the non-linearity of this relationship to observe the variation in the child mortality under five in the second stage. In view of the earlier studies on the squared effect of population density on air pollution (PM_2.5_) in the atmosphere, we also additionally control for the square of population density. The result is negative but not significant. The coefficient of the constant in Table 2 shows the average severe magnitude of particulate matter in the atmosphere of sampled countries.

Table 3 column (1) shows the 2SLS country fixed effect result of particulate matter PM_2.5_ (endogenous) on the log of child mortality across countries after integrating the income effect with PM_2.5_. More specifically, a one-unit annual increase in PM_2.5_ is likely to increase the mean of child mortality under five in sampled countries by 14.5%, with a 1% significance level controlling for the potential effect of population density (WHO dataset). This is striking given the mean number of children dying before the age of five (e.g., mean = 39,924, as per WHO dataset) in the studied countries shown in Table A1. Given the significant effect of PM_2.5_ on child mortality across countries, when we control for average education, the partial effect of PM_2.5_ becomes insignificant, suggesting the potential influence of average education on the attitude of people towards environmental pollution. Columns (3) and (4) in Table 3 shows the effect of endogenous PM_2.5_ on child mortality under five years in the sampled countries. Specifically, the annual increase by one unit in air pollution (PM_2.5_) is likely to increase the average mortality of children under five by 15.2% (WB dataset). After controlling for education, the result is not significant, which is consistent with the idea of education’s effect on peoples’ behavior.

**Figure A1:**
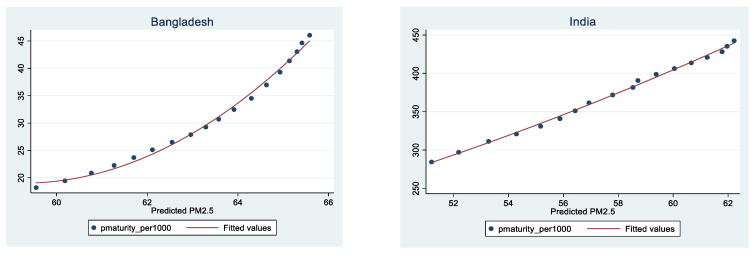

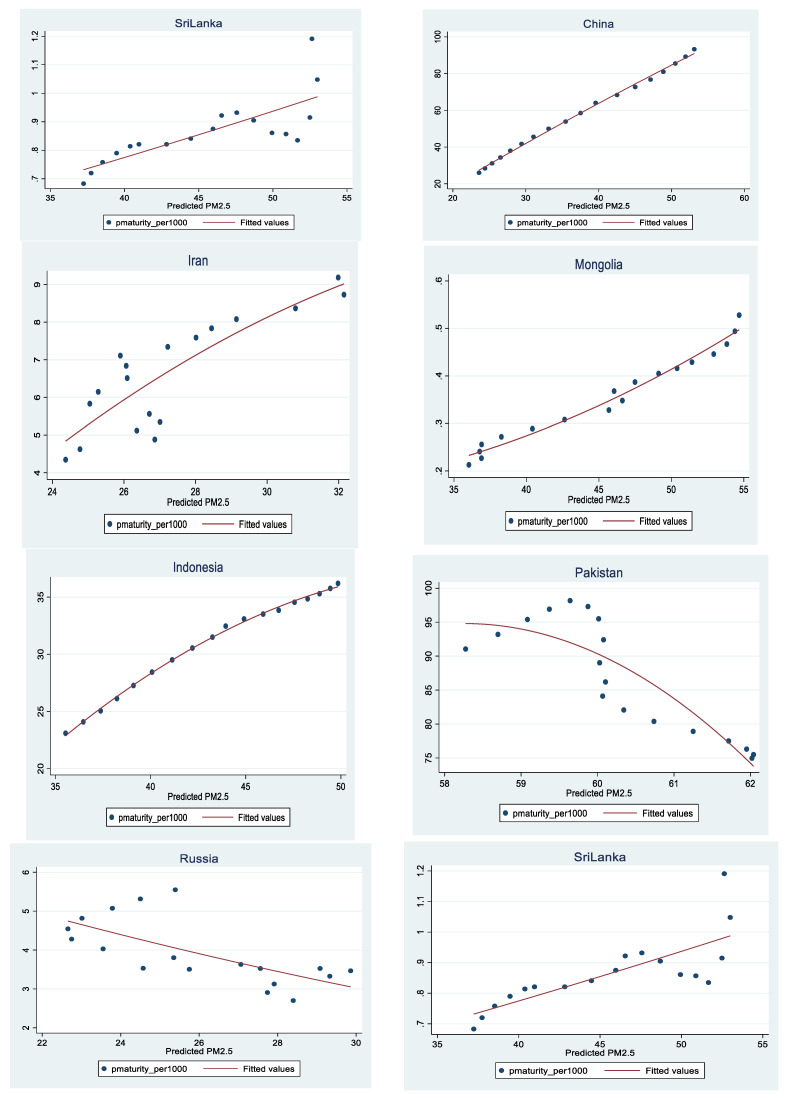

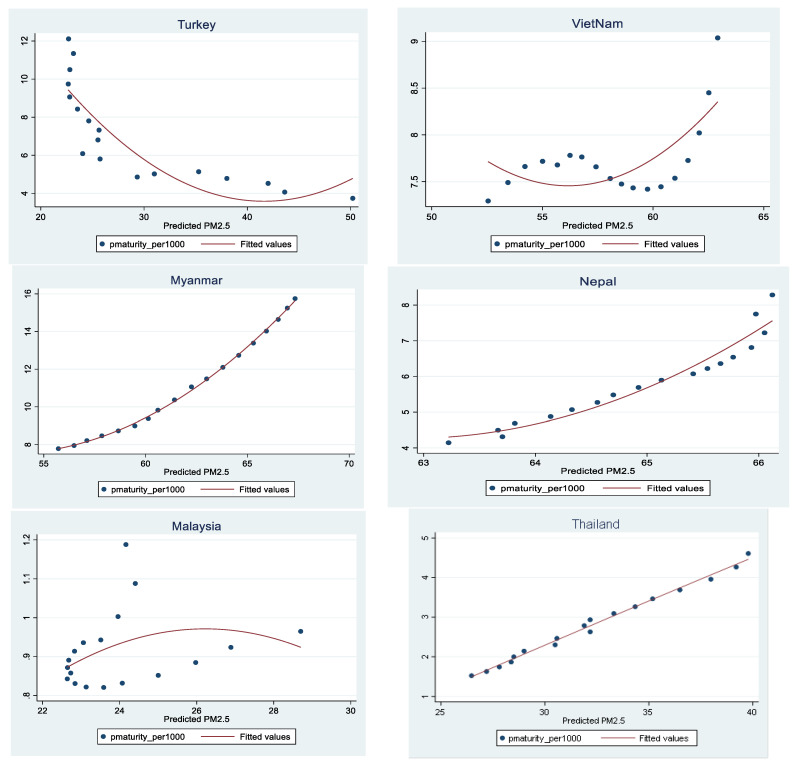
Predicted PM_2.5_ effect on child mortality in Asian countries. **Note:** The above figures show the relationship between the predicted values of PM_2.5_ and child mortality in each country. The *y*-axis represents child mortality while the *x*-axis represents predicted PM_2.5._ The values of predicted PM_2.5_ are obtained through regressing PM_2.5_ on income, and the square of income. The upward-trending quadratic fitted line in most of the sampled countries is consistent with our aggregate second-stage results shown in Table 3, except for Turkey, Russia and Pakistan. We show this relationship in Figure A1 for all countries in our case. We observed that for most of the countries, our second-stage results are consistent, showing a positive effect of PM2.5 on child mortality. For some countries, this effect may not be observed in the Figures; however, as we use the conditional mean as the best linear unbiased estimator, any differences observed in individual countries do not falsify the inference inferred on average. We additionally control for the idiosyncratic behaviors of countries by using the country fixed effect in our estimation.

We show the relationship between the predicted values of PM_2.5_ and child mortality in each country in Figure A1. The upward-trending quadratic fitted line in most of the sampled countries is consistent with our aggregate second-stage results shown in Table 3 except for Turkey, Russia and Pakistan. We observed that for most of the countries, our second-stage results are consistent, showing a positive effect of PM_2.5_ on child mortality.

## 4. Discussion

This study examined the relationship between air pollution (PM_2.5_) and child mortality in sixteen Asian countries over the period of 2000 to 2017. Our results are striking. Following an alternate model of two-stage least squares (2SLS), we found sizable 14.5% and 15.2% relationships between income and child mortality under five in both the WHO and WB datasets triggered by PM_2.5_ in the sixteen sampled countries, suggesting a transitional stage of these economies in terms of development. The high growth in these economies through industrialization has increased the combustion of energy resources such as coal, fuel and natural gas, with these being important resources for the economy’s production [5,11]. The thrust towards achieving higher economic growth in the sampled countries is resulting in deteriorated environmental conditions, pushing increased air pollution (PM_2.5_) and consequent serious health threats. Our results are corroborated by the studies of [5,15,16], who highlighted a significant relationship between air pollution (PM_2.5_) and child mortality. The studies in India, China [20,27], and other developing countries [3,13,28] have also established a significant relationship between air pollution and health outcomes. Some studies found that the incidence rates of child morbidity and mortality are higher among children living in highly polluted areas [29]. The WHO reports 2.3 billion people in Asian Pacific countries living with air pollution at levels considered harmful for human health. The environmental performance index developed by Columbia and Yale university shows Asia’s unsatisfactory performance, i.e., second lowest EPI score, which is just higher than Sub-Saharan Africa. The low performance in terms of environmental health is causing higher risks for the population in Asia, who are already subjected to polluted air and water, and excessive metal exposure. The continuing pace of economic growth and urbanization in Asian countries will result in even worse health outcomes due to air pollution as a result of poor resources and a lack of advanced technologies. Therefore, green finance is an environmentally friendly solution to the pollution faced by Asian countries.

Air pollution is responsible for one out of every eight deaths globally (WHO, 2016). Air pollution affects child mortality in different ways. During the gestation period, children’s lungs and immune systems are at the development stage, and therefore, are more vulnerable to air pollution [30,31]. Lelieveld et al. [1] reported that 96% of global childhood mortality is caused by air pollution and consequent lower respiratory infection (AAP-LRIs). One of the principal causes behind children’s vulnerability to LRIs is twofold, i.e., a higher breathing rate and oral inhaling, causing children to take in more air polluted with particulate matters, thus triggering lower respiratory infections [3,32]. Moreover, PM_2.5_ particles are very small in size, and can easily bypass the natural filters present in children’s nostrils, causing respiratory infections and consequent mortality [3].

Other associated factors include preterm birth and low birthweight, stimulating child mortality under five. The WHO (2016) attested that 1 million children die every year due to problems of preterm birth. Studies have shown that mothers exposed to pollutants experience premature births and low birth weights in their babies [33,34]. An alarming feature pushing preterm births is mounting air pollution. The countries with the highest numbers of preterm births are facing severe air pollution. Air pollution affects the pregnant mothers directly and indirectly, e.g., when the mother inhales polluted air containing CO_2_ and other pollutants, these pollutants mix between their bloodstream and the fetus, which has a direct effect on the baby’s health. A similar phenomenon was tested and proven by [35], who conducted studies on pregnant women exposed to particulate matters, and found carbon particles in placental samples taken after delivery of these fetuses.

The results further show that the average education decreases the severity of pollutants’ effects on child mortality. This is evident from adding average education as a control variable, as shown in Table 3. Education does not only help in increasing economic growth and individual’s incomes, but it also helps in developing behaviors and habits that contribute positively to an individuals’ health [36]. Educated parents have a greater ability to provide better care to their children, and make the best use of the health and other social services accessible to them. Education helps parents to make good and timely decisions about different health and disease factors, such as basic hygiene, prenatal care, immunization and nutrition, which are important in reducing the leading causes of child deaths under five.

Child mortality under five years has been studied in this article, but pollutants other than PM_2.5_, such as O_3,_ NO_2_ and SO_2_, have not been investigated. The overall effect of these pollutants could be larger, and this requires further investigation. In addition to the two datasets, other datasets can be used to compare the results of the impact of air pollution on child mortality in those under five in future studies.

## 5. Conclusions

This study examined the impact of air pollution (PM_2.5_) on child mortality in sixteen Asian countries during 2000 to 2017. The study used data on child mortality as measured by the number of children dying before reaching the age of five (WHO and WB (per 1000 live births) data sets), air pollution measured (in terms of PM_2.5_) mean annual exposure (micrograms per cubic meter), education and population density. We adopted a two-staged least square model and explored variation in PM_2.5_ due to increasing economic growth. We found a non-linear relation of economic growth with PM_2.5_, suggesting a U-shaped income–environment relationship in Asian countries. Controlling for socio-economic covariates, our 2SLS fixed effect model found a significantly positive effect of PM_2.5_ on child mortality under five. When we controlled for average education, the effect of PM_2.5_ became insignificant, suggesting an important role of education in explaining the aggregate behavior of the population towards children’s health. Our results are also consistent in explaining the effect of PM_2.5_ on child mortality under five (WB dataset) in the sampled countries.

The results of this study will provide insights encouraging policy makers to admit the relation of children mortality to air pollution (PM_2.5_) and formulate effective policies to improve air quality for the general public at large. In this case, the governments of Asian countries may not wait for the long-term effects of income on PM_2.5_. Alternate policies, such as green financing and environmentally friendly technologies that reduce PM_2.5_ emissions, as well as further education programs and strategic urbanization policies to evenly distribute population density, should be implemented to overcome the severity of air pollution-related health effects. Moreover, the financial sector should focus on green investment opportunities and programs, such as green bonds, green home and building loans, and go green auto loans, to achieve green status and lower pollution in these countries.

## Figures and Tables

**Table 1:** Reduced-form effect of PM_2.5_ on child mortality.

	Log of Child Mortality (WHO Dataset)		Log of Child Mortality (WB Dataset)	
	(1)	(2)	(3)	(4)
Log of PM_2.5_	−0.0984	−0.0885	−0.176	−0.168
	(−1.047)	(−0.821)	(−1.392)	(−1.093)
Log of GDP Per Capita	1.079	0.961	1.152	1.058
	(1.414)	(1.351)	(1.289)	(1.305)
Log of (GDP Per Capita)^2^	−0.121 **	−0.104 **	−0.126 **	−0.110 *
	(−2.441)	(−2.209)	(−2.306)	(−2.088)
Log of Population Density	−0.356	−0.403	−1.458	−1.486
	(−0.388)	(−0.449)	(−1.533)	(−1.542)
Log of (Population Density)^2^	0.0330	0.0551	0.0577	0.0709
	(0.389)	(0.582)	(0.661)	(0.758)
Average Education		−0.00556 **		−0.00538 **
		(−2.239)		(−2.148)
Constant	9.399 **	9.267 ***	8.236 **	8.130 **
	(2.886)	(3.290)	(2.292)	(2.460)
Country FE	YES	YES	YES	YES
Observations	160	159	160	159
R-squared	0.745	0.769	0.853	0.874
Number of Countries	16	16	16	16

Note: Table 1 shows the reduced-form results for the effect of covariates on child mortality. Standard errors are clustered at country-level. Unbalanced panel data from 2000 to 2017 are used for 16 Asian countries. The dependent variable in columns (1) and (2) is the log of total number of children dying under five (WHO dataset). As expected, it appears from the coefficients of covariates in columns (1) and (2) that after controlling for the square of GDP and population density, the effect of PM_2.5_ is statistically not significant. This means that the presence of GDP and population in the same regression with PM_2.5_ potentially underestimates the effect of PM_2.5_ on the outcome variable, strengthening the possibility that these two variables might have indirect effects on health, e.g., through PM_2.5_, which requires running regression in two stages. The dependent variable in columns (3) and (4) is the log of child mortality under five (per 1000 live births) (WB). Columns (3) and (4) show that GDP and the square of GDP have no direct effect on health. In using both datasets, the direct health effect of income and population is not significant. Therefore, in the two-stage least square model, we attempt to see the indirect effect (e.g., the effect of income and population on health through PM_2.5_). Values in brackets represent z-values. *** *p* < 0.01, ** *p* < 0.05, * *p* < 0.1.

**Table 2:** First-stage estimation for air pollution (PM_2.5_).

Dep. Var: PM_2.5_	OLS	OLS-FE	OLS RE
			
GDP Per Capita	−0.0105 *	−0.00279 ***	−0.00314 ***
	(−1.990)	(−3.109)	(−3.491)
(GDP Per Capita)^2^	6.23 × 10^−7^ *	1.24 × 10^−7^ **	1.39 × 10^−7^ ***
	(1.840)	(2.648)	(2.886)
Population Density	0.0164	0.00626	0.0239
	(0.228)	(0.134)	(0.624)
(Population Density)^2^	−6.90 × 10^−6^	−4.57 × 10^−6^	−1.14 × 10^−5^
	(−0.132)	(−0.227)	(−0.641)
Constant	65.91 ***	51.21 ***	49.03 ***
	(3.497)	(6.588)	(9.313)
F-Test	15.85	12.79	-
R-squared	0.349	0.117	-
Observations	160	160	160

Note: the countries included in this study are Bangladesh, China, India, Indonesia, Iran, Malaysia, Mongolia, Myanmar, Nepal, Pakistan, Philippines, Russia, Sri Lanka, Thailand, Turkey and Vietnam. Standard errors are clustered at the country level. The dependent variable PM_2.5_ is measured in terms of mean annual exposure (micrograms per cubic meter). Unbalanced panel data from 2000 to 2017 are used for 16 Asian countries. Values in brackets represent T-values in the first two columns and Z-values in the last column. *** *p* < 0.01, ** *p* < 0.05, * *p* < 0.1.

**Table 3:** Second-stage estimation on child mortality.

	Child Mortality (WHO Dataset)		Child Mortality (WB Dataset)	
	(1)	(2)	(3)	(4)
PM_2.5_	0.145 ***	0.0935	0.152 **	0.1000
	(2.760)	(1.568)	(2.460)	(1.463)
Population Density	−0.000218	0.00415	−0.00514	−0.00154
	(−0.0255)	(0.553)	(−0.567)	(−0.191)
(Population Density)^2^	−8.28 × 10^−7^	−2.57 × 10^−6^	1.25 × 10^−6^	−1.65 × 10^−7^
	(−0.225)	(−0.806)	(0.323)	(−0.0490)
Average Education		−0.0109		−0.0109
		(−1.392)		(−1.140)
Constant	2.742	5.054	−2.492	−0.0135
	(3.253)	(3.343)	(3.695)	(3.718)
Country FE	YES	YES	YES	YES
Observations	160	159	160	159
Number of country1	16	16	16	16

Note: Standard errors are clustered at country level. Unbalanced panel data from 2000 to 2017 are used for 16 Asian countries. The endogenous variable is the air pollution (PM_2.5_) estimated through GDP per capita and the square of GDP per capita. The dependent variable in columns (1) and (2) is the log of total number of children dying before the age of five (WHO datasets). The dependent variable in columns (3) and (4) is the log of child mortality under five per 1000 live deaths (WB dataset). Data are obtained from the WHO and World Bank tables. Values in brackets represent z-values. *** *p* < 0.01, ** *p* < 0.05.

## Data Availability

The data presented in this study are available on request from the corresponding author.

## Appendix A

**Table A1:** Descriptive Statistics.

Variable Description	Observations	Mean	Standard Deviation	Min	Max	Source
Total Number of Premature Deaths (WHO)	288	39,924.17	89,881.62	213	442,544	World Health OrganizationGlobal Health Repository
Premature Child Mortality per 1000 live births (WB data)	288	36.13	25.81	7.4	112.6	Work Bank Tables
PM2.5 (Particulate Matter)	160	44.1774	24.51686	11.09962	100.7844	Work Bank Tables
GDP Per Capita	288	3805.489	3454.672	346.7747	14,936.4	Work Bank Tables
Population Density	288	223.1709	265.3905	1.543189	1265.036	Work Bank Tables
Mortality (Less than 5 years)	288	36.13681	25.81633	7.4	112.6	Work Bank Tables
Average Education	287	72.92878	20.52273	20.01234	120.6316	Work Bank Tables
